# Analysing the Antibacterial Synergistic Interactions of Romanian Lavender Essential Oils via Gas Chromatography–Mass Spectrometry: In Vitro and In Silico Approaches

**DOI:** 10.3390/plants13152136

**Published:** 2024-08-01

**Authors:** Roxana Aurelia C. Bălașoiu (Jigău), Diana Obistioiu, Anca Hulea, Mukhtar Adeiza Suleiman, Iuliana Popescu, Doris Floares (Oarga), Ilinca Merima Imbrea, Alina-Georgeta Neacșu, Laura Șmuleac, Raul Pașcalău, Laura Crista, Cosmin Alin Popescu, Ionel Samfira, Florin Imbrea

**Affiliations:** 1 Faculty of Agriculture, University of Life Sciences “King Michael I” from Timisoara, Calea Aradului 119, 300645 Timisoara, Romania; roxana.jigau@usvt.ro (R.A.C.B.); anca.hulea@usvt.ro (A.H.); iuliana_popescu@usvt.ro (I.P.); doris.oarga@usvt.ro (D.F.); alinaneacsu@usvt.ro (A.-G.N.); laurasmuleac@usvt.ro (L.Ș.); raul.pascalau@usvt.ro (R.P.); laura.crista@usvt.ro (L.C.); cosmin_popescu@usvt.ro (C.A.P.); ionelsamfira@usvt.ro (I.S.); florin_imbrea@usvt.ro (F.I.); 2 Faculty of Life Science, Department of Biochemistry, Ahmadu Bello University, Zaria 810107, Nigeria; masuleiman@abu.edu.ng; 3 Faculty of Engineering and Applied Technologies, University of Life Sciences “King Michael I” from Timisoara, Calea Aradului 119, 300645 Timisoara, Romania; ilinca_imbrea@usvt.ro

**Keywords:** *Lavandula angustifolia* Mill. cv. “*Vera*”, *Lavandula angustifolia*, cv. “*Sevtopolis*”, *Lavandula × intermedia* “*Grosso*” *(lavandin)*, lavender essential oil, GC-MS, antioxidant activity, antibacterial activity, antibiotic enhancement, molecular docking

## Abstract

This study investigated the phytochemical characteristics, antibacterial activity, and synergistic potential of essential oils derived from Romanian lavender. Gas Chromatography–Mass Spectrometry (GC/MS) analysis revealed that linalool is the main compound in all lavender essential oils, with concentrations ranging from 29.410% to 35.769%. Linalyl acetate was found in similar concentrations to linalool. Other significant compounds included 1,8-cineole (8.50%), lavandulyl acetate (5.38%), trans-β-ocimene (6.90%), and camphor (7.7%). A 1,1-Diphenyl-2-Picrylhydrazyl (DPPH) test was used to assess antioxidant capacity, with substantial free-radical-scavenging activity shown in the IC_50_ values determined. The antibacterial efficacy of the oils was higher against Gram-positive bacteria than Gram-negative bacteria, with variations in minimum inhibitory concentrations (MICs), the extent of inhibition, and evolution patterns. The study also explored the oils’ ability to enhance the efficacy of ampicillin, revealing synergistic interactions expressed as fractional inhibitory concentration indices. In silico protein–ligand docking studies used twenty-one compounds identified by GC-MS with bacterial protein targets, showing notable binding interactions with SasG (−6.3 kcal/mol to −4.6 kcal/mol) and KAS III (−6.2 kcal/mol to −4.9 kcal/mol). Overall, the results indicate that Romanian lavender essential oils possess potent antioxidant and antibacterial properties, and their synergistic interaction with ampicillin has potential for enhancing antibiotic therapies.

## 1. Introduction

Nature represents a rich source of biologically active compounds that can be used as alternative treatment methods to combat various pathologies to the detriment of synthetic compounds. From the past to the present, from primitive formulations to complex combinations, aromatic and medicinal plants have been used as a natural source of remedies and healthcare [1]. Lavender is one of the most popular medicinal plants, known for its anti-parasitic, antiviral, antioxidant, anti-inflammatory, antibacterial, and anticancer effects [2,3,4,5,6,7]. Originating from Mediterranean areas, over 30 lavender cultivars belonging to the Lamiaceae family are known. Due to its biological activity, the most important variety is narrow-leaved lavender [2]. However, its biomedical attributes are correlated with its chemical composition, which varies depending on the climate, the geographical area, the stage of development and the harvest period, and not least, the method of extraction of the phytocompounds [8,9].

Lavender essential oil (LEO), extracted by different methods from the plant’s flowering tops, is a promising candidate with antibacterial activity [7,10,11], an important aspect considering that, recently, a worrisome problem worldwide is the emergence of multidrug-resistant bacteria to most antibiotics used in medical practice [12]. This biological activity is attributed mainly to the monoterpene content of linalool and linalyl acetate, found in variable concentrations in different geographic areas [7,11,13,14]. Predoi et al. [14] found that LEO from Southern Romania had a concentration of 47.55% β-linalool, while Todorova et al. [15] found a concentration of 27.67% of the same monoterpene in Bulgarian LEO. LEO from Southern Africa is characterised by the predominance of linalyl acetate (36.7%), with linalool being the second most abundant compound (31.4%) [16]. Other monoterpenes identified frequently in LEO from different countries, in various concentrations, besides linalyl acetate, are represented by camphor, borneol, ocimene, and terpinene [7,11,13,14]. Due to different concentrations of monoterpene content, biological activities are varied.

Excellent antibacterial activity is correlated with a high, almost equal concentration of linalool and linalyl acetate, and a ratio of the sum of linalyl acetate and linalool to terpinen-4-ol of more than 13 [17]. However, antibacterial activity correlates with the chemical composition of the essential oil (EO) and the type of bacteria tested. Although data in the literature claim that LEO has antibacterial activity against both Gram-positive and Gram-negative strains [18], most have demonstrated that Gram-positive ones are more susceptible [15,19,20]. The different sensitivity between the two groups of bacteria can be attributed to variations in their cell wall structure and composition. Compared to Gram-positive bacteria, Gram-negative ones have an outer membrane that contains lipopolysaccharides, which may further impede the penetration of active substances into bacterial cells [21]. Among the Gram-positive bacterial strains that have been proven to be sensitive to LEO in the literature are *Staphylococcus aureus* (MRSA), *Staphylococcus epidermidis*, *Streptococcus mutans*, *Bacillus cereus*, *Listeria monocytogenes*, and *Enterococcus faecalis* [18,19,20,22]. *Aeromonas* spp., *Escherichia coli*, *Bordetella bronchiseptica*, *Shigella sonnei*, *Salmonella abony*, *P. mirabilis*, and *S. enterica* represent the Gram-negative bacteria sensitive to LEO [22,23,24].

Regarding the sensitivity of *Pseudomonas aeruginosa,* this is debatable. The study carried out by Hossain et al. [23] demonstrates that *P. aeruginosa strains* are resistant [23], while other studies show that LEO has antibacterial activity against this bacterial strain, even if in increased concentration [18,25]. Overall, the values of minimum inhibitory concentrations are between 2.5 and 10 mg/mL, with variations from one bacterial strain to another [7,18]. Moreover, in addition to its antibacterial activity when used alone, LEO synergises with other EOs or antibiotics, enhancing its therapeutic effect. Kwiatkowski et al. [7] demonstrated the synergic antibacterial activity of LEO with octanin dihydrochloride against methicillin-resistant *Staphylococcus aureus*. A binary combination of *Lavandula cfu* and *Origanum majorana* displayed a partial synergistic effect against the same bacterial strain [26]. The synergistic activity of LEO with peppermint was highlighted by Angane et al. [27] when the antibacterial capacity of this combination against foodborne pathogens was tested [27]. When added to gentamicin, LEO has a synergic antibacterial effect against *S. aureus* and no interaction against *P. aeruginosa* [28].

Bacteria, in their very core of existence, are opportunistic in their nature of colonisation. An adhesion to various cell-wall-anchoring proteins often follows this colonisation. *Staphylococcus aureus* Surface protein G (SasG) is one of these proteins, contributing excellently to *S. aureus*’s adhesion to human skin [29,30]. Another essential protein is the β-ketoacyl acyl carrier protein (ACP) synthase III (KAS III), which is involved directly or indirectly in the pathogenesis of common inflammatory skin diseases [31]. A molecular docking study to show how the compounds in lavender oil interact with these two proteins is necessary to further elucidate the mechanism by which the oil can inhibit their function in bacteria. Additionally, the docking results will attest to the relevance of the binding affinity, complex stability, and binding interaction (hydrogen and hydrophobic) occurring between the proteins (PDB: 6A9N, 8G1M) and the identified compounds in lavender oil. The knowledge herein will reveal the potential interaction of natural products with the protein structures of microorganisms, thereby aiding the chemotherapeutic understanding of their antibiotic relevance [32].

Considering the versatility of lavender oil and the variability influenced by its chemical composition and by bacterial strains, the research objectives were as follows: (i) to evaluate the chemical composition of Romanian LEOs using Gas Chromatography–Mass Spectrometry (GC/MS); (ii) to evaluate radical-scavenging activity by conducting a 1,1-Diphenyl-2-Picrylhydrazyl (DPPH) assay and determine the IC_50_ values of antioxidant activity; (iii) to assess the antibacterial activity of three LEOs against various microbial strains; (iv) to evaluate the potential of each LEO to potentiate the antibacterial activity of Ampicillin, an antibiotic belonging to the aminopenicillin class of the penicillin family; and (v) to analyse the molecular docking between the chemical components and the main proteins found in the bacterial wall. Ampicillin is an antibacterial agent approved by the Food and Drug Administration for clinical use, testing, and reporting by microbiology laboratories included in Group A. This an-tibacterial agent is deemed to be appropriate for inclusion in routine, primary testing panels, as well as for the routine reporting of results.

The study highlights the special qualities of essential lavender oils made from plants from Romania, adding new information to the knowledge base about lavender’s phytochemical and pharmacological profiles. Although EOs derived from lavender have previously been investigated, this study offers an in-depth examination of the chemical composition of Romanian lavender, highlighting oxygenated monoterpenes such as linalool and linalyl acetate as significant components. By testing LEOs against various microbial strains, the research expands our understanding of the antibacterial capabilities of lavender oils. The investigation of the binding interactions between Romanian EO compounds and bacterial protein targets (SasG and KAS III) through in silico docking studies is a novel approach, and the study’s findings on fractional inhibitory concentration indices suggest a novel way to enhance antibiotic efficacy, potentially addressing antibiotic resistance issues. The docking scores offer insight into the underlying mechanisms of LEO by providing a molecular foundation for the observed antibacterial activity.

## 2. Results

### 2.1. GC/MS Analysis Results

The results of the chemical characterisation by GC/MS are presented in Table 1.

All three LEOs tested were characterised by increased concentrations of oxygenated monoterpenes between 69.280 and 82.282%. The concentration of monoterpene hydrocarbons was almost equal to the one of sesquiterpene hydrocarbons in the cases of L.A.V. (12.71% vs. 11.830%) and L.H.G. (6.894% vs. 6.313%), and almost a quarter of the concentration in the case of L.A.S. (13.59% vs. 3.720%). From the oxygenated monoterpenes, increased concentrations of linalool were distinguished for all the tested oils, with values between 29.410% and 35.769%. In comparison, increased concentrations of linalyl acetate were identified only in the cases of L.A.V. (26.810%) and L.H.G. (35.319%). L.A.S. was characterised by an increased concentration of endo-borneol (10.44%), while for the other two EOs, this oxygenated monoterpene was under 0.5% or undetectable. From the monoterpene hydrocarbons, trans-beta-ocimene was detected as the majority compound (2.830–6.900%), followed by cis-beta-ocimene in the case of L.A.S. (3.510%) and L.H.G. (1.833%), and D-Limonene (1.850%) in the case of L.A.S.

The predominant sesquiterpene hydrocarbons (SHs) in all samples were cis-beta-Farnesene (2.330–5.460%) and beta-caryophyllene (0.750–5.290%). Only two were detected. Concerning oxygenated sesquiterpenes (SOs), caryophyllene oxide was present in all the LEOs tested.

### 2.2. Antioxidant Activity by 1,1-Diphenyl-2-Picrylhydrazyl (DPPH) Assay

To assess radical-scavenging activity using the DPPH method, five concentrations (100 mg/mL, 50 mg/mL, 25 mg/mL, 12.5 mg/mL, and 10 mg/mL) of each EO dissolved in methanol were prepared from the three samples tested. Concurrently, the antioxidant activity of five ascorbic acid solutions at varying concentrations (0.006–0.016 mg/mL) was also measured as a positive control, with the highest concentration (0.016 mg/mL) showing 91.13% inhibition (Table 2). The IC_50_, the concentration of each EO required to cause 50% DPPH inhibition, was calculated and expressed in mg/mL (Table 3).

According to the values presented in Table 1, the highest radical-scavenging activity was observed at the maximum concentration (100 mg/mL) for all samples. Additionally, the highest percentage of inhibition belonged to L.H.G. (69.51%), followed by L.A.V. (65.78%) and L.A.S. (60.52%).

The statistical program JASP. 0.18.3 used the variance analysis (ANOVA) method to test the existence of significant differences between the means of the data groups. The values are presented in Table 3.

In the present study, the total variability of the data was divided into two components in terms of the expression of DPPH inhibition in the case of the three LEOs. Thus, the variation between groups (due to treatment) was tested, as was the influence of the five concentrations (100 mg/mL, 50 mg/mL, 25 mg/mL, 12.5 mg/mL, and 10 mg/mL) on the variation in the antioxidant capacity of the three LEOs (L.A.S., L.A.V., and L.H.C.).

To analyse the significance of the *p*-value, we compared the F-values determined with the help of the critical values in the previous distribution table. Thus, in this case, the significance value of *p* was <0.001, which refutes the null hypothesis and statistically assures us of a distinctly significant interdependence between the means of the groups.

To perform the post hoc analysis, we used Tukey’s test to determine the difference between the response means of the three LEOs. Thus, the determination of the degree of “Honest Significant Difference” in Table 4 shows that a significant differentiation between the percentages of inhibition belongs to L.H.G. (69.51%), followed by L.A.V. (65.78%) and L.A.S. (60.52%).

The IC_50_ values (Table 5) varied, with the L.H.G. sample showing the highest antioxidant capacity at 4.10 mg/mL. In contrast, the L.A.V. and L.A.S. samples demonstrated lower antioxidant capacities at 4.47 mg/mL and 4.71 mg/mL, respectively.

### 2.3. The Antibacterial Activity of LEOs

Table 6 presents the BIR% values obtained according to Formula (3) presented in 4.5. The BIR% values are expressed as percentages reported to the positive controls.

L.A.V., L.A.S., and L.H.G. presented similar patterns concerning the Gram-positive and Gram-negative strains affected, the difference being in the inhibitory values or, in the case of *P. aeruginosa* and *S. flexneri*, the different positive BIR% values (Table 6).

L.A.V. presented an ascending evolution in a positive correlation with the increase in concentration for all Gram-positive bacteria tested, and all Gram-negative bacteria except for *P. aeruginosa* and *S. flexneri*. *S. pyogenes* and *B. cereus* showed similar MICs of 0.5 mg/mL and BIR% values of 2.83% and 5.95%. *S. aureus* demonstrated an MIC of 1 mg/mL, while *L. monocytogenes* and *C. perfringens* showed inhibition only at the highest concentration tested of 4 mg/mL, which was demonstrated by inhibitory values of 0.64% and 4.04%. Gram-negative bacteria proved more resistant to the antibacterial activity, but this was proven only in *E. coli* (4 mg/mL MIC), *S. typhimurium* (1 mg/mL MIC), and *H. influenzae* (4 mg/mL MIC). *P. aeruginosa* and *S. flexneri* demonstrated a negative trend, with efficacy decreasing alongside the increase in concentration and the EO components presenting synergism with the bacterial strains, providing a strain-boosting effect.

Regarding the activity of L.A.S. against Gram-positive bacteria, the picture is one of lower efficacy than L.A.V, with decreasing MICs, but still an ascending evolution positively correlated to the increase in concentration. The Gram-negative bacteria showed resistance concerning *P. aeruginosa* and *S. flexneri*, with no MICs and negative correlation, depicting a strain-boosting activity, while *E. coli*, *S.typhimurium*, and *H. influenzae* showed similar inhibitory thresholds to L.A.V.

L.H.G. also showed inhibitory efficacy on the strains tested, but at higher MICs, with only 2 mg/mL or 4 mg/mL values. The resistance trends of *P. aeruginosa* and *S. flexnery* were also present, similar to L.A.V. and L.A.S.

### 2.4. Evaluation of the Capacity to Potentiate the Antibacterial Activity of Ampicillin

The results of the synergy test revealed the bacterial strains’ high sensitivity to the enhanced effect of the EOs and ampicillin in a high percentage (Table 7). Therefore, the L.A.V/ampicillin mixture demonstrated synergistic values (FICI ≤ 0.5) for all Gram-positive bacteria and *S. typhimurium*, and no interaction in the case of *E. coli* and *H. influenzae*. The L.A.S./ampicillin mixture demonstrated no interaction with *S. pyogenes*; all the other strains tested (except *P. aeruginosa* and *S. flexneri,* which demonstrated resistance to ampicillin) demonstrated FICI values ≤ 0.5. LH.G./ampicillin demonstrated the best FICI values, with only *S. pyogenes* (FICI = 2.125) demonstrating no interaction. The other difference between the mixtures, besides the lower FICI values, was the presence of an FICI value in the case of *S. flexneri*, even though the value obtained suggested no interaction (FICI = 1.01).

### 2.5. Molecular Docking

The docking of the lavender oil compounds against the SasG protein showed lavandulyl acetate, acetic acid-hexyl ester, 3 octanone, nerol-acetate, octen-1-ol, acetate, linalool acetate, linalool, and eucalyptol to have binding affinity to the protein with free binding energies of −5.8 kcal/mol to −4.6 kcal/mol. Similarly, linalool acetate, acetic acid-hexyl ester, lavandulyl acetate, 3-octanone, nerol acetate, octen-1-ol, and acetate also had the best binding affinity to KAS III, with free binding energies of −6.3 kcal/mol to −4.9 kcal/mol (Table 8).

**Figure 1:**
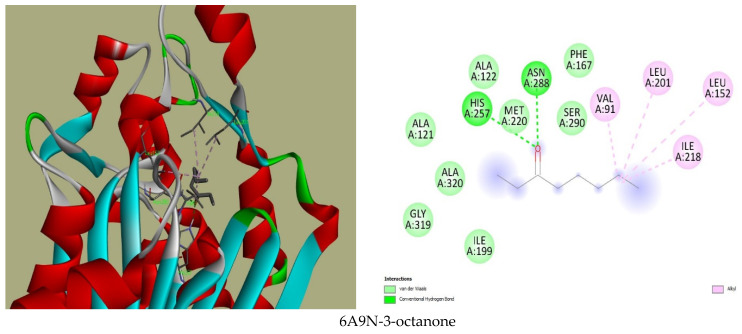

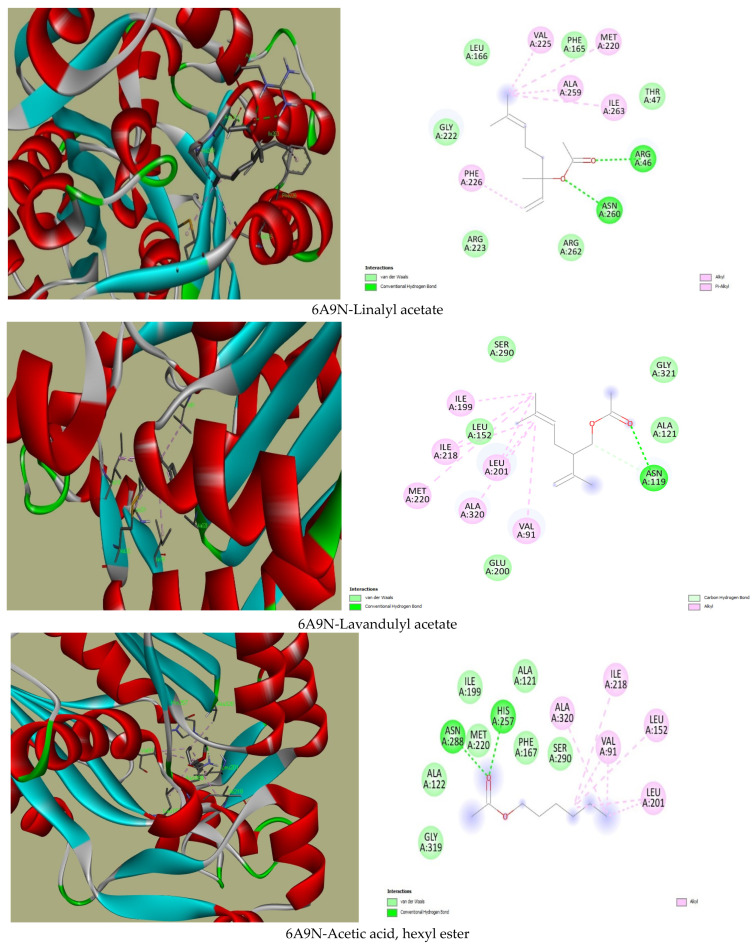

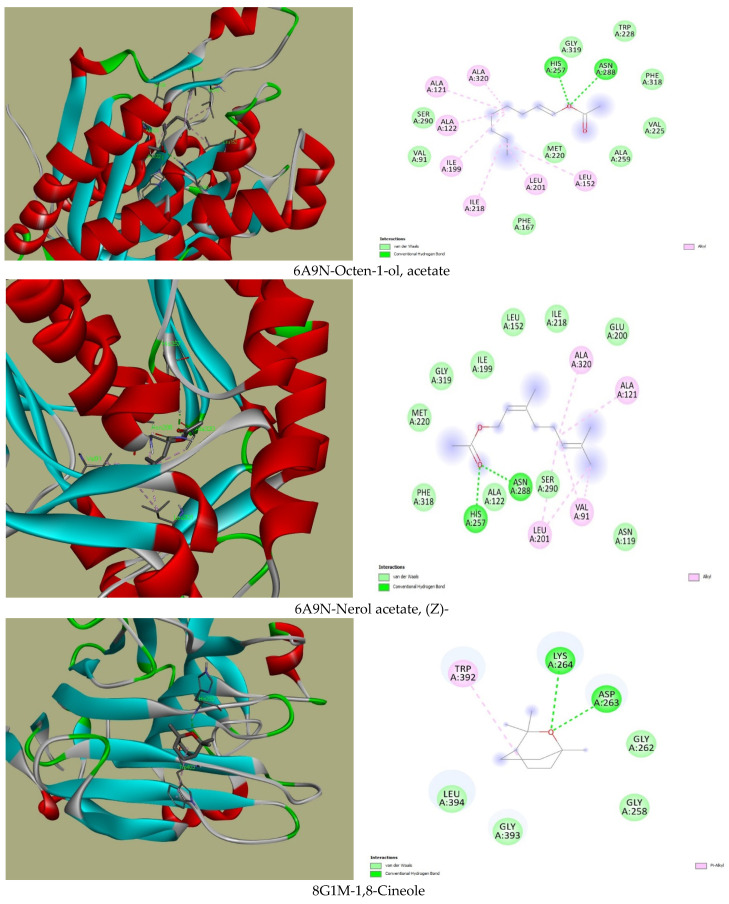

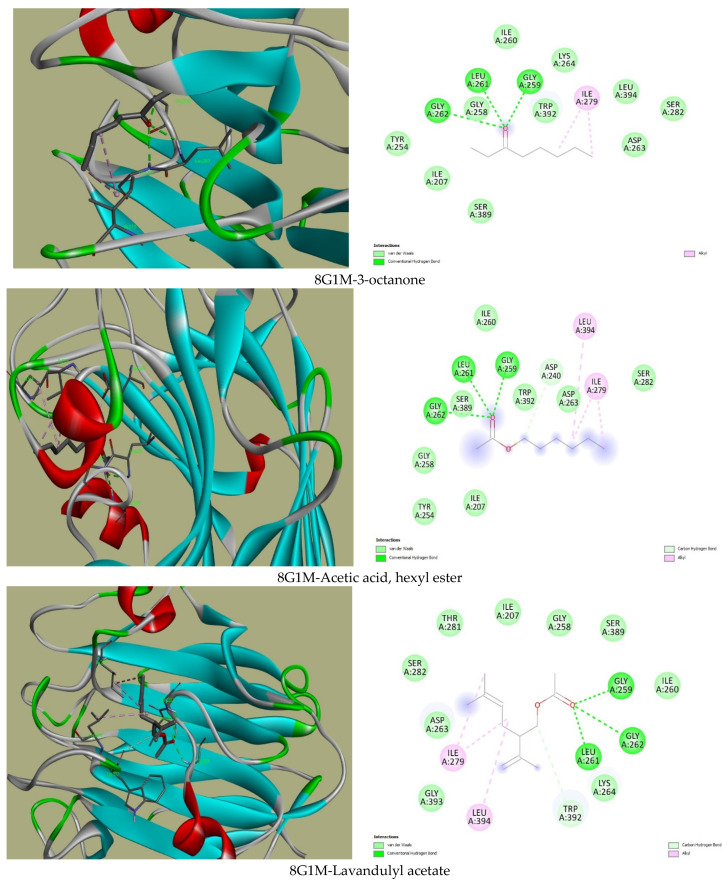

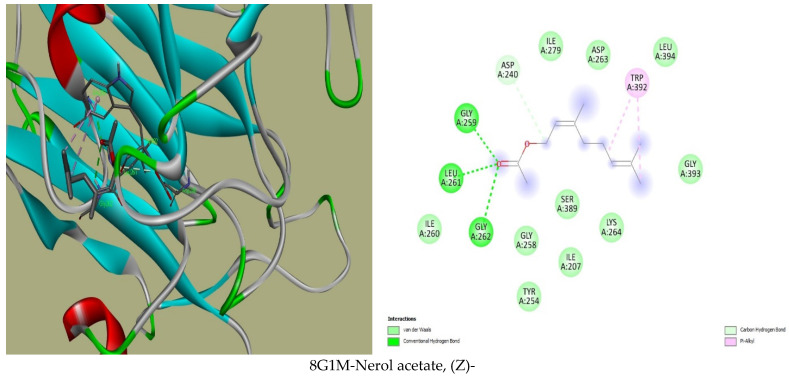
Three-dimensional and two-dimensional binding interactions between the compounds of lavender oil with the β-ketoacyl acyl carrier protein (ACP) synthase III (KAS III) and the lectin domain of *Staphylococcus aureus* surface protein G (SasG).

The visualisation of the receptor–ligand (protein–compound) interaction revealed interesting complexes stabilised by H-bond interactions and other hydrophobic bonds. Among these compounds, the interactions between lavandulyl acetate and acetic-hexyl ester with the SasG protein showed three H-bonds, one carbon–hydrogen bond, and three alkyl bonds, respectively. Particularly, the hydrogen bonds were formed with amino acid residues GLY259, GLY262, and LEU261, the carbon–hydrogen bonds were with ASP240 and TRP392, and the alkyl bonds were mostly observed with ILE279, LEU 394, and TRP392. On the other hand, the interactions of the binding complex between the compounds and KAS III showed two hydrogen bonds and several alkyl bonds. The hydrogen bonds formed with residues ARG46, HIS257, ASN 260, ASN288, and SER290, while the alkyl bonds were with VAL91, LEU152, LEU201, and ILE218 (Figure 1). This further establishes these proteins’ sites as active targets of the compounds from the oils.

## 3. Discussion

### 3.1. Gas Chromatography–Mass Spectrometry (GC/MS)

EO compounds are derived from two biosynthetic groups: terpenes (monoterpenes, sesquiterpenes, and their derivatives) and phenylpropanoids. LEO is characterised by the following predominant monoterpenes: linalool, linalyl acetate, geraniol, lavandulol, borneol, bornyl acetate, terpineol, 1.8-cineole, and champhor. However, the concentration of chemical compounds in EO is influenced by several factors, such as the variety and the geographical area where the plant grew. Jeddi et al. [33] state that the volatile oil obtained from the Taounate region, Mernissa, contains linalool (28.94%), linalyl acetate (19.95%), and caryophyllene (6.46%) as its major constituents. *L. angustifolia* EOs from Ukraine contained linalool and linalyl acetate as their main components, ranging from 11.4% to 46.7% and 7.4% to 44.2%, respectively. The third most abundant component was terpinen-4-ol in a concentration of 1–19% [34]. In contrast, Kozuharova et al. [35] demonstrated that the major constituents of the EO samples from an agricultural plantation near Pomorie, Bulgaria, were linalyl acetate (27.5%) and linalool (24.1%), with the third most abundant compound being E-β-ocimene (7.0%), followed by terpinen-4-ol (5.1%) [35]. Similarly, Todorova et al. [15] demonstrated that a *Lavandula angustifolia* EO from the Thracian Lowland, Bulgaria, was characterised by a high concentration of linalool (20.0–45.0%) and linalyl acetate (20.79–39.91%). Commercial samples also contained linalool and linalyl acetate, as described in the European Pharmacopoeia [15]. According to Marchidan et al. [36], *L. angustifolia* EO from Romania contains high concentrations of linalool (39.10–40.38%) and eucalyptol (14.59–19.51%) and low concentrations of linalyl acetate (6.40–5.41%). In contrast, Bogdan et al. [10] demonstrated that Romanian *L. angustifolia* Mill EO is rich in linalool and linalyl acetate, with values 3.5–27.39% for linalool and 26.60–40.66% for linalyl acetate. Similarly, the present study showed that the main compound in all LEOs is represented by linalool, in different concentrations, ranging between 29.410% and 35.769%. Even though all the EOs were similar in terms of linalool, differences were remarked regarding other major compounds. Linalyl acetate was identified in approximately equal concentrations with linalool in the case of L.H.G. and L.A.V., while for L.A.S., a low concentration of linalyl acetate was detected (5.97%). 1,8 cineole (8.50%) was the second most abundant oxygenated monoterpene for L.A.S., while for the other two the concentrations were only around 1%. The third major compound identified was lavandulyl acetate (5.38%) for L.H.G., trans-β- ocimene (6.90%) for L.A.V., and camphor (7.7%) for L.A.S. Overall, even though all the EOs were characterised by high concentrations of oxygenated monoterpenes, L.H.G. had the highest concentration (82.282%) and L.A.S. the lowest (69.280%). Regarding the sesquiterpene hydrocarbons contained, differences were also observed between the tested oils. The highest concentrations were detected in L.A.V. (11.830%) and the lowest in L.A.S. (3.720%). From this class of terpenes, cis-beta-Farnesene (2.330–5.460%) was the majority representative for all EOs. Also, beta-caryophyllene was detected in almost the same concentrations as cis-beta-farnesene in the case of L.A.V., while in the case of L.H.G., it was under 1%. These variations in the concentrations of the different monoterpene compounds in the LEOs studied are justified by the various cultivars and explain the variable biological activity. However, even if it is known which compounds are responsible for certain biological effects (for example, that linalool and linalyl acetate have antibacterial and antioxidant activity [33,37,38]), the biological activity of each oil is actually the result of the synergistic or antagonistic interaction of all the oil’s compounds.

### 3.2. Antioxidant Activity by 1,1-Diphenyl-2-Picrylhydrazyl (DPPH) Assay

The antioxidant activity of EOs is attributed to their polyphenol compounds, due to their ability to prevent the formation of free radicals from endogenous processes and intervene with exogenous factors [39].

The present study demonstrated that LEOs can scavenge DPPH free radicals, even though all the values were lower than with ascorbic acid. However, the percentage inhibition values were dose-dependent, with the maximum values observed at the highest concentration tested (100 mg/mL). The inhibition values ranged between 6.38 and 60.52% for L.A.S., 8.99 and 65.78% for L.A.V., and 10.23 and 69.51% for L.H.G. Similarly, Marchidan et al. documented a 68.14% inhibition in the EO of *Lavandula angustifolia* from Romania when examining its antioxidant, antibacterial, antifungal, and antiproliferative properties [36]. Moreover, Hadj Moussa et al. [40] reported a maximum inhibition value of 67.06% at 100 mg/mL of *Lavandula angustifolia* EO from Seraïdi, Northeastern Algeria. In contrast, *L. angustifolia* EO from Iraq showed higher antioxidant activity, with a percentage inhibition of 88.91% at 1000 μg/mL [41].

By calculating the IC_50_ of antioxidant activity, the present study demonstrated that the values are around 4 mg/mL for all *Lavanda* EOs tested, as follows: 4.47 mg/mL for L.A.V. mg/mL, 4.71 mg/mL for L.A.S., and 4.10 mg/mL for L.H.G. Due to the fact that the IC_50_ values were close to each other for all the EOs, differences were not noted regarding antioxidant activity. This indicated that all the lavender EOs tested had better antiradical activity than others from other world regions. For example, in a previous study, lavender EO from Croatia had an IC_50_ of 27.67 mg/mL [42], while one of Italian origin had a value of 28.57 mg/mL. Similarly, El Abdaly et al. [43] reported IC_50_ values of 12.95 ± 1.30 mg/mL in 2022 for the antioxidant activity of Moroccan lavender, while Jeddi et al. [33] sustained that Moroccan volatile oil from *L. angustifolia* had an IC_50_ of 197.69 ± 9.22 μg/mL. Kıvrak [44], in her analysis of the composition and antioxidant activities of eight lavender and lavandin cultivars from western Anatolia, found inhibition values of 23.67 μg/mL for the *L. angustifolia* Yubileina cultivar and 89.81 μg/mL for the *L. x intermedia* Super A cultivar. However, few data from the literature demonstrate LEOs’ potent antioxidant activity. Nikšić et al. [45] observed an IC_50_ value of 0.421 mg/mL in their study of *Lavandula angustifolia* Mill, while Carrasco et al. [46] obtained an IC_50_ value of 1.4 µg/mL and demonstrated better activity when the oils were extracted by distillation than hydrodistillation.

All this demonstrates that, similar to other pharmacological effects, antioxidant activity is influenced by extraction methods and the geographical region influencing the plant’s chemical composition. Antioxidant activity is associated with the presence and position of hydroxyl groups in polyphenol molecules [39]. Linalool (2,6-dimethyl-2,7-octadien-6-ol) is a monoterpenoid with a methyl group at positions 3 and 7, and the hydroxyl group found at position 7 possesses strong antioxidative properties [47,48]. Other compounds known as antioxidant substances are 1,8-cineole (eucalyptol), terpinolene, and phellandrene [49,50].

### 3.3. The Antibacterial Activity of LEOs

Similar to other biological effects, antibacterial activity is influenced by the chemical composition of oils, as well as their terpenoid composition. Phenolic compounds strongly inhibit microbial growth, while terpene alcohols and esters present slightly weaker antibacterial activity [51].

LEO contains higher concentrations of monoterpenes, mainly linalool and linalyl acetate, which are compounds recognized for their significant antibacterial activity by disrupting the integrity and function of the bacterial wall. The mechanism of linalool action seems to be increasing membrane permeability, which determines losses of ions and cellular constituents [52,53]. Similarly, linalyl acetate affects bacterial walls, causing the leakage of intracellular materials by perturbing the lipid fraction of bacterial plasma membranes [54]. However, the synergic action of these compounds, and others identified in low concentrations, is critical for creating a unique mixture resulting in antibacterial activity [7]. Even though antibacterial effects were demonstrated against most Gram-positive and Gram-negative bacteria, other MIC values reported in the literature are variable, depending on the composition of the LEOs and the bacterial strain tested [18,19,20,22,55]. Broadly, the literature supports a higher efficiency against Gram-positive bacteria than Gram-negative bacteria [18,19,20,22], an aspect also observed in the present study. El Hachlafi et al. [37] showed that concentrations of 0.015–0.25% *v*/*v* of LEO are efficient against Gram-positive bacteria, while for Gram-negative ones, the MIC values are between 0.125 and 0.5% *v*/*v*, while Jeddi et al. [33] demonstrated that the MIC value against some Gram-negative bacteria can reach even 1% *v*/*v*. In our study, the MIC values obtained were between 0.5 mg/mL and 4 mg/mL for Gram-positive bacteria, while most of the Gram-negative ones were between 2 mg/mL and 4 mg/mL, with variations depending on the type of EO and bacterial strain tested. The differences between the two types of Gram bacteria, regarding the structure of the bacterial wall, can explain the different MIC values, which were higher for Gram-negative and lower for Gram-positive ones.

*S. aureus* is known as one of the most critical pathogens due to its ability to achieve antibiotic resistance [56]. The present study showed that a concentration between 0.5 mg/mL and 2 mg/mL can inhibit bacterial growth. Similarly, other studies have demonstrated that the MIC value of LEO against *S. aureus* is around 2 mg/mL [57,58]. According to the literature, other Gram-positive pathogens also seem sensitive to LEO in different concentrations [18,58]. Among those mentioned are *Enterococcus faecalis*, *Micrococcus luteus*, *Bacillus subtilis*, *Bacillus cereus* [18], and *Listeria monocytogenes*. According to Walasek-Janusz et al. [18], the MIC value against *B. cereus* is between 2.5 mg/mL and 5 mg/mL. These results, which are contradictory with ours, demonstrated that lower concentrations (0.5–4 mg/mL) of Romanian LEO are capable of inhibiting the bacterial growth of this strain. Similarly, antibacterial activity against *L. monocytogenes* was observed at a concentration between 1 mg/mL and 4 mg/mL, despite other studies sustaining that more than 100 µL/mL is necessary to inhibit bacterial growth [59].

The present study demonstrated that the LEOs tested are efficient against Gram-negative bacteria in various concentrations. Of all of them, *S. typhimurium* had the lowest MIC value (1–2 mg/mL). In contrast, the growth of *S. flexneri* was not inhibited in the presence of L.A.S. or L.A.V., while only L.A.S. had no antibacterial activity against *P. aeruginosa*. However, the MIC values of L.A.V. and L.A.S. against *P. aeruginosa* were 2 mg/mL and 4 mg/mL, respectively. This remark contradicts the result obtained by Walasek-Janusz et al. [18], who sustained that inhibiting the growth of *S. typhimurium* and *P. aeruginosa* strains requires higher oil concentrations: 5–10 mg/mL for *S. typhimurium* and 10 mg/mL for *P. aeruginosa* [18]. In contrast, Denkova et al. [57] showed that a concentration of only 500 µg/cm^3^ is necessary to inhibit the growth of *P. aeruginosa* and *E. coli*. However, the present study highlighted that the *E. coli* strain has MIC values of 2 mg/mL in the case of L.H.G. and 4 mg/mL using L.A.V. and L.A.S., similar to those observed for *H. influenzae*. On the other hand, Puvaca et al. [58] sustained that LEO has no antibacterial activity against *E. coli* and *S. typhimurium*. These differences observed in the literature sustain the hypothesis that every EO obtained by different methods and from regions around the world comprises a mixture of phyto-compounds that, through their synergistic or antagonistic action, results in unique biological characteristics.

All three oils demonstrated similar patterns regarding the types of bacterial strains affected, with higher efficacy against Gram-positive bacteria than Gram-negative bacteria. Variations were primarily found in the minimum inhibitory concentration (MIC), the extent of inhibition, and different responses to *P. aeruginosa* and *S. flexneri*. Regarding L.A.V.’s activity against Gram-positive bacteria, the oil exhibited ascending inhibitory effectiveness with increased concentration, and its MIC values were 0.5 mg/mL for *S. pyogenes* and *B. cereus*; 1 mg/mL for *S. aureus*; and 4 mg/mL for *L. monocytogenes* and *C. perfringens*. Concerning the Gram-negative bacteria, the MIC values were 4 mg/mL for *E. coli*, 1 mg/mL for *S. typhimurium*, and 4 mg/mL for *H. influenzae*. Also, a negative trend was demonstrated for *P. aeruginosa* and *S. flexneri*, indicating reduced efficacy with higher concentrations and potential strain-boosting effects. L.A.S. demonstrated lower efficacy than L.A.V., but showed increasing inhibition with higher concentrations and similar MIC values to L.A.V. for most strains. Concerning Gram-negative bacteria, L.A.V. demonstrated resistance to *P. aeruginosa* and *S. flexneri*, showing no inhibitory effects and a negative correlation, but presented similar inhibitory thresholds to L.A.V. for *E. coli, S. typhimurium*, and *H. influenzae*. Concerning L.H.G.’s antibacterial effects against Gram-positive and Gram-negative bacteria, the oil showed inhibitory efficacy at higher MICs (2 mg/mL or 4 mg/mL), while *P. aeruginosa* and *S. flexneri* exhibited resistance trends similar to those observed with L.A.V. and L.A.S.

### 3.4. Evaluation of the Capacity to Potentiate the Antibacterial Activity of Ampicillin

Potentiation, or synergism, is a more significant response than expected when two or more drugs are mixed. For more than a century, the concept of synergistic interactions between medications and substances has been a significant concern in the biomedical field. The idea of finding new synergic interactions between substances is sustained by the fact that many diseases require a mixture of various drugs for treatment [60]. Moreover, for infectious diseases with bacterial aetiology, identifying the synergism between two substances with antibacterial activity has particular importance since, in recent decades, an inefficiency in therapy has been observed due to the emergence of multidrug-resistant strains [61,62,63].

The literature demonstrates that LEO can potentiate the antibacterial activity of other EOs or synthetic substances [7,27,55,64]. Kwiatkowski et al. [7] showed that LEO presented synergistic activity in combination with OCT (octenidine dihydrochloride) against methicillin-resistant *S. aureus* strains, with the values of FICI ranging from 0.11 to 0.26. Adaszyńska-Skwirzyńska et al. [28] found a synergistic effect against *S. aureus* strains when LEO was combined with gentamicin, with values of FICI between 0.076 and 0.320. However, when studying the impact of this mixture against *P. aeruginosa*, no interaction was observed (FICI = 1.083–1.300). Another study demonstrated that addition (FICI = 0.5–1) was observed when a mixture of lavender oil and enrofloxacin was used against *Salmonella* spp. [64]. Moreover, the synergism of this mixture (FICI = 0.115–0.5) has frequently been demonstrated in tests of antibacterial activity against *E. coli*, and only in a few cases has addition been observed [65]. The present study demonstrated the synergistic activity of all three LEOs tested when mixed with ampicillin against *L. monocytogenes*, *B. cereus*, *C. perfringens*, and *S. typhimurium*. Against *E. coli* and *H. influenzae,* only the associations of L.A.S. + ampicillin and L.H.G. + ampicillin generated an FICI value under 0.5.

Regarding the synergistic effects with ampicillin, the L.A.V./ampicillin mixture developed synergistic effects (FICI ≤ 0.5) for all Gram-positive bacteria and *S. typhimurium*, and had no interaction with *E. coli* or *H. influenzae*. As for the L.A.S./ampicillin mixture, no interaction with *S. pyogenes* was observed, while synergistic effects for other strains were recorded, except for *P. aeruginosa* and *S. flexneri*. The L.H.G./ampicillin mixture presented the best FICI values overall, with only *S. pyogenes* (FICI = 2.125) showing no interaction.

The synergistic action of lavender oil with various antibiotics could represent an advantage in therapies for bacterial infections by decreasing the concentration of the active substance and implicitly increasing the therapeutic efficiency. Moreover, these mixtures could become effective even against resistant bacteria.

### 3.5. Molecular Docking

The adoption of virtual screening aimed at the possible exposition of the protein–ligand interaction has been shown to be an alternative way to identify biologically relevant targets for discovering new anti-pathogenic drugs [66]. Molecular docking studies are growing to complement other multivariate analyses, allowing for the further examination of lead compounds acting as protein inhibitors with possessive antibacterial activity. The skin is a defined niche for commensal bacteria such as *Staphylococcus* species, which bridge against the pathogens already on the skin. *Staphylococcus aureus* surface protein G (SasG) is reported to promote adhesion to shedded epithelial cells, as well as flakes of dead cells [67]. The β-ketoacyl acyl carrier protein (ACP) synthase III (KAS III) is an attractive antibacterial target, with its key function expressed in fatty acid synthesis [68].

The antibacterial effects of the major compounds identified from the GC-MS of the three lavender oils show the formation of stable complexes between the compounds and the crystal structures of two bacterial proteins with binding affinities that corroborate hydrogen and other hydrophobic interactions. In the docking in the current study, the oxygenated monoterpenes (1,8-cineole, lavandulyl acetate, linalool, linalyl acetate, and nerol acetate) recorded fascinating binding interactions (hydrogen bonds) with SasG and KAS III proteins (Figure 1). This is possibly because of the hydroxyl and oxo-acid groups in these compounds, which allow for reasonably observed interactions. This is attested by the hydrogen bonds that formed with amino acid residues GLY259, LEU261, and GLY262 of SasG, in addition to the hydrophobic interactions, most notably of TRP392, reported on the floor of the glycan binding pocket of the protein. Ultimately, these binding modes will cause the stacking of interactions between the compounds and the lectin of the SasG protein [69]. Cumulatively, the involvement of HIS257, ALA259, ASN260, and ASN288 in the hydrogen bond with KAS III connotes their reported function in the catalytic triad of the protein and in acyl group transfer from acyl-CoA and malonyl-ACP [68,70]. Specifically, these interactions could support the blockage by the compounds in lavender oil and in the amino acid residues of KAS III.

## 4. Materials and Methods

### 4.1. Chemicals

All reagents used for chemical analysis were purchased from Sigma–Aldrich Chemie GmbH (München, Germany) and Geyer GmbH (Renningen, Germany), and were of analytical quality.

### 4.2. Samples of LEO

All samples were obtained directly from private producers of organic lavender cultivars on the western side of Romania who grow and process lavender for EO for the market. The EOs were obtained by the steam distillation of the flowering top parts of the fresh lavender plant material collected in 2023. All lavender plants were 3–7 years old.

To ensure the correctness of the experimental design, voucher specimens were botanically identified and deposited in a temperature-controlled herbarium (22–25 °C and 30–40% relative humidity) in the Botany Department at the University of Life Sciences King Michael I of Romania in Timişoara, with the codes VSNH.ULST-BD92, VSNH.ULST-BD93, and VSNH.ULST-BD94. The lavender species used to obtain EOs were confirmed as *Lavandula angustifolia* Mill. cv. “*Vera*” (L.A.V.), *Lavandula angustifolia*, cv. “*Sevtopolis*” (L.A.S.), and *Lavandula × intermedia* “*Grosso*” (L.H.G.).

The EOs tested in the laboratory were clear, pale-yellow liquids with prominent floral and spicy aromas. All the EOs exhibited a floral scent; however, L.A.V. and L.A.S. had a more pronounced herbal odour, while a fresher aroma characterized L.H.G.

### 4.3. Gas Chromatography–Mass Spectrometry (GC/MS)

The EOs were determined by a gas chromatograph (Shimadzu2010, Kyoto, Japan) coupled with a triple-quadrupole mass spectrometer (TQ 8040, Shimadzu, Kyoto, Japan) and a Zebron ZB-5plus (30 m × 0.25 mm i.d., 0.25 µm film thickness, Phenomenex, Torrance, Canada, Torrance, CA, USA). The carrier gas used was He, with a 1 mL min^−1^ flow. The oven temperature was initially 70 °C; this was held for 11 min, before being raised to 190 °C at a rate of 5 °C min^−1^ and then to 240 °C at a rate of 20 °C min^−1^, where it was kept for 5 min. The injector and MS source temperatures were set to 250 °C and 200 °C, respectively. The injection volume was 1 µL, with a split ratio of 10:1. Before the injection, the EO samples were diluted (1:100, *v*:*v*) with hexane. All the samples were filtered using a 0.45 µm PTFE membrane. All chemical constituents were identified using spectra libraries NIST 14 and Wiley 09 [71]. Using normal alkane RI for the same polar column, the LRI (Linear Retention Index) was calculated [72]. The values obtained expressed the percentage area of the chromatographic bands (peaks) on the chromatogram, which corresponded to the compounds identified.

### 4.4. Antioxidant Activity by 1,1-Diphenyl-2-Picrylhydrazyl (DPPH) Assay

The evaluation of antioxidant capacity through the DPPH method was conducted following the procedure outlined by Floares et al. [73]. To assess the antioxidant properties of EO, a methanolic sample was prepared by dissolving 1 mL of EO in 10 mL of methanol (Sigma–Aldrich; Merck KGaA, Darmstadt, Germany). The extracts were refrigerated at 2–4 °C until the analysis.

Different concentrations (10, 12.50, 25, 50, and 100 mg/mL) were prepared by diluting the base extract. First, 3 mL of each dilution was mixed with 1 mL of 0.3 mM DPPH solution and then incubated in darkness for 30 min. The absorbance of the samples was measured at 517 nm using a UV-VIS spectrophotometer (Specord 205; Analytik Jena AG, Jena, Germany). Each sample was tested in triplicate, and the average was reported.

A control experiment was performed simultaneously, replacing the extract with methanol and maintaining the same volume and concentration of DPPH solution. Ascorbic acid (0.016 mg/mL in methanol) from Lach-Ner Company (Neratovice, Czech Republic) was used as a positive control.

The antioxidant activity was quantified as the percentage of radical-scavenging activity (RSA) using the following Formula (1):

RSA (%) = (A_control_ − A_sample_)/(A_control_) * 100 (1)

where A_control_ is the absorbance value of the control sample and A_sample_ is the absorbance value of the EO sample.

The antioxidant capacity of the sample was expressed as the IC_50_ value and compared to that of ascorbic acid.

### 4.5. Evaluation of Antibacterial Activity

The antibacterial activity of the LEOs used in the study was evaluated by broth microdilution against Gram-positive and Gram-negative ATCC strains.

The Gram-positive ATTC strains tested were *Streptococcus pyogenes* (ATCC 19615), *Staphylococcus aureus* (ATCC 25923), *Clostridium perfringens* (ATCC 13124), *Listeria monocytogenes* (ATCC 19114), and *Bacillus cereus* (ATCC 10876). The Gram-negative strains studied were *Pseudomonas aeruginosa* (ATCC 27853), *Shigella flexneri* (ATCC 12022), *Escherichia coli* (ATCC 25922), *Salmonella typhimurium* (ATCC 14028), and *Haemophilus influenzae tip B* (ATCC 10211). All the ATTC bacterial strains are part of the Laboratory of Microbiology culture collection at the Interdisciplinary Research Platform within the University of Life Sciences, “King Mihai I of Romania”, Timisoara.

Our previous studies describe the method [74]. Briefly, a stock solution of EO was prepared in dimethylsulphoxide (DMSO), and then 50 µL of stock solution containing different LEOs was added over 100 µL freshly grown bacterial suspension diluted at an optical density (OD) of 0.5 by the McFarland standard (1.5 × 10^8^ CFU/mL). Serial dilutions were performed.

All three lavender essentials, L.A.V., L.A.S., and L.H.G., were tested at different concentrations: 0.0075 mg/mL, 0.0150 mg/mL, 0.0300 mg/mL, 0.0600 mg/mL, 0.1250 mg/mL, 0.2500 mg/mL, 0.5000 mg/mL, 10 mg/mL, 20 mg/mL, and 40 mg/mL.

A pure, uninhibited strain grown in brain heart infusion broth was used as a positive control.

The MIC, the lowest compound concentration that yields no visible microorganism growth, was determined by the measurement of OD using the spectrophotometric method, according to [75].

Two indicators were calculated for interpreting the results—BGR (bacterial growth rate) and BIR (bacterial inhibition rate)—according to Formulas (2) and (3):

BGR = OD_sample_/OD_negative control_ × 100 (%) (2)

BIR = 100 − BGR (%)(3)

where OD_sample_ represents the optical density of the sample, containing a certain type and concentration of the EOs tested at 540 nm as a mean value of triplicate readings, and OD_negative control_ is the optical density at 540 nm as a mean value of triplicate readings for the selected bacteria in BHI.

### 4.6. Evaluation of the Capacity to Potentiate the Antibacterial Activity of Amoxicillin

The interaction between LEOs and amoxicillin was tested using the checkerboard method, as previously described in the literature [55]. Briefly, into each well of a 96-well plate, 100 µL of a 1.5 × 10^8^ CFU/mL (0.5 McFarland standard) bacterial suspension was inoculated. The antibacterial actions of L.A.V., L.A.S., L.H.G., and ampicillin (a series of two-fold dilutions made in the range of 0.125 ÷ 32 µg/mL) (Sigma-Aldrich, code A5354, Merck KGaA, Darmstadt, Germany) mixtures were determined using the minimum inhibitory concentration (MIC) microdilution assay and verified according to CLSI recommendations (Clinical and Laboratory Standard Institute, 2002). The dilutions were performed using DMSO (POCH, Gliwice, Poland). Clinical and Laboratory Standard Institute (CLSI) recommendations were followed [76,77].

The ampicillin concentration, selected according to each MIC obtained, was mixed with 50 µL of DMSO containing different concentrations of the LEOs tested. Then, the plates were incubated for 24 h at 37 °C in aerobic conditions. All tests were performed in triplicate.

After the incubation period, the OD of each well plate was measured using the spectrophotometric method, according to [75].

The bacterial suspensions contained the same ATTC strains used to evaluate the antibacterial activity of the LEOs.

The FICI (fractional inhibitory concentration index) was calculated using the following equations:

FIC_EO_ = MIC_value of EO combined with Ciprofloxacin_/MIC_value of EO alone_ (4)

FIC _Ciprofloxacin_ = MIC_value of Ciprofloxacin combined with EO_/MIC_value of Ciprofloxacin alone_ (5)

FICI = FIC_value of EO_ + FIC_value of Ciprofloxacin_ (6)

The results were interpreted as follows: synergism (FICI < 0.5), addition (0.5 ≤ FICI ≤ 1.0), indifference (1.1 < FICI ≤ 4.0), or antagonism (FICI > 4.0).

### 4.7. Molecular Docking Studies

To assess the possible binding affinity of twenty-one (21) compounds in lavender oil to the two bacterial proteins, PyRx virtual screening software [78] was adopted. The PDB files for each protein were obtained from [79] (PDB ID: 6A9N and 8G1M). The SDF files of the compounds were retrieved from [72]. The protein crystal structures were prepared in the PDBQT format for docking using AutoDock to make the loaded macromolecule and the imported compounds. Two- and three-dimensional interactions were visualised in Discovery Studio v21.1.0.20298 (BIOVIA, San Diego, CA, USA).

### 4.8. Statistical Analysis

Statistical analysis was performed using JASP 0.18.3. Descriptive statistics, including mean and standard deviation, were calculated to evaluate the study data. Group comparisons were conducted using analysis of variance (ANOVA), followed by the Tukey test for post hoc analysis. The threshold for statistical significance was set at *p* < 0.05.

## 5. Conclusions

This study underlines Romanian lavender essential oils’ significant phytochemical diversity and bioactivity, highlighting their potential as organic antioxidants and antibacterial agents. Strong antioxidant characteristics, increased antibacterial efficacy against Gram-positive bacteria, and encouraging synergistic effects with ampicillin are significant aspects. Subsequent investigations should concentrate on extensive phytochemical assessments, intricate mechanistic analyses, the investigation of possible synergistic antibiotic combinations, in vivo validations, and formulation development. Clinical trials and regulatory safety assessments are also required for medical and consumer products to determine safe use and therapeutic potential.

## Figures and Tables

**Table 1:** Chemical composition of the LEOs detected by GC-MS.

		RT* (min)	RI*	L.A.V.	L.A.S.	L.H.G.	ISO 3515:2002 [15]
	**Monoterpene hydrocarbons**			**12.710**	**13.590**	**6.894**	
1	Allo-Ocimene	3.385	850	0.45 ± 0.01	0.14 ± 0.02	nd	
2	alpha- Terpinolene	14.341	1088	nd	0.58 ± 0.01	nd	
3	alpha-Pinene	6.027	939	0.24 ± 0.01	0.77 ± 0.02	nd	
4	alpha-Thujene	5.739	930	0.12 ± 0.01	0.14 ± 0.01	nd	
5	beta- Myrcene	8.132	978	nd	nd	0.56 ± 0.01	
6	beta-Phellandrene	11.073	1025	0.18 ± 0.01	4.14 ± 0.02	0.22 ± 0.03	Max 1%
7	beta-Pinene	8.357	979	0.56 ± 0.02	0.83 ± 0.03	nd	
8	Camphene	6.667	954	0.12 ± 0.01	0.56 ± 0.03	nd	
9	cis-beta-Ocimene	11.393	1037	3.51 ± 0.01	1.04 ± 0.03	1.83 ± 0.02	0.5–6%
10	D-Limonene	10.896	1020	0.32 ± 0.01	1.85 ± 0.03	0.34 ± 0.01	Max 1%
11	gamma-Terpinene	12.812	1059	0.16 ± 0.01	0.28 ± 0.01	nd	
12	o-Cymene	10.570	1019	nd	nd	0.15 ± 0.04	
13	p-Cymene	9.493	990	0.15 ± 0.01	0.20 ± 0.01	nd	
14	Sabinene	7.596	975	0.05 ± 0.01	0.23 ± 0.03	nd	
15	trans-beta-Ocimene	12.089	1050	6.90 ± 0.03	2.83 ± 0.02	3.77 ± 0.01	1–10%
	**Oxygenated Monoterpenes**			**69.280**	**77.260**	**82.281**	
16	1,8-Cineole	11.145	1029	1.35 ± 0.08	8.50 ± 0.05	0.76 ± 0.03	Max 3%
17	alpha-Terpineol	19.463	1196	0.85 ± 0.01	0.63 ± 0.02	0.97 ± 0.01	Max 2%
18	Camphor	17.439	1146	0.29 ± 0.01	7.70 ± 0.09	0.20 ± 0.01	
19	cis- Linalool oxide	16.393	1123	nd	nd	0.22 ± 0.01	
20	cis-Geraniol	20.532	1232	0.08 ± 0.01	0.04 ± 0.01	nd	
21	endo-Borneol	18.586	11.69	0.49 ± 0.01	10.44 ± 0.04	nd	
22	Lavandulol	18.170	1168	1.04 ± 0.01	0.65 ± 0.03	nd	
23	Lavandulyl acetate	22.575	1292	4.42 ± 0.05	0.95 ± 0.01	5.38 ± 0.04	Max 8%
24	Linalool	15.460	1096	29.41 ± 0.06	35.59 ± 0.05	35.76 ± 0.02	20–43%
25	Linalyl acetate	21.454	1258	26.81 ± 0.04	5.97 ± 0.02	35.31 ± 0.02	25–47%
26	Nerol acetate	19.093	1180	0.22 ± 0.01	0.07 ± 0.01	0.35 ± 0.05	
28	Terpinen-4-ol	18.906	1177	4.32 ± 0.02	6.72 ± 0.03	3.28 ± 0.03	Max 8%
	**Sesquiterpenes hydrocarbons SH**			**11.830**	**3.720**	**6.312**	
29	alpha-Humulene	27.712	1454	0.14 ± 0.01	0.10 ± 0.01	nd	
30	alpha-Santalene	28.870	1491	nd	nd	0.35 ± 0.05	
31	beta-Caryophyllene	26.726	1419	5.29 ± 0.05	0.75 ± 0.05	2.16 ± 0.02	
32	cis-alpha-Bergamotene	27.062	1414	0.14 ± 0.01	0.07 ± 0.02	nd	
33	cis-beta-Farnesene	27.590	1414	5.46 ± 0.07	2.33 ± 0.01	3.44 ± 0.03	
34	gamma-Cadinene	29.225	1512	0.11 ± 0.01	0.02 ± 0.01	nd	
35	Germacrene D	28.408	1481	0.69 ± 0.01	0.45 ± 0.01	0.35 ± 0.06	
	**Oxygenated sesquiterpenes SO**			**0.230**	**0.570**	**0.340**	
36	alpha-Bisabolol	33.437	1687	nd	0.47 ± 0.02	nd	
37	Caryophyllene oxide	31.014	1583	0.23 ± 0.01	0.10 ± 0.01	0.34 ± 0.03	
	**Oxygenated aliphatics OA**			**3.080**	**1.290**	**4.163**	
38	1-Hexanol	4.125	873	0.07 ± 0.01	0.28 ± 0.01	nd	
39	1-Octen-3-yl-acetate	15.659	1105	0.70 ± 0.02	0.01 ± 0.01	1.19 ± 0.03	
40	3-Octanol	8.783	982	0.21 ± 0.01	0.01 ± 0.02	nd	
41	3-octanone	9.493	990	nd	nd	0.99 ± 0.01	Max 3%
42	Acetic acid, hexyl ester	9.673	1010	1.00 ± 0.02	0.10 ± 0.03	0.62 ± 0.04	
43	Butanoic acid, hexyl ester	19.328	1194	0.82 ± 0.02	0.72 ± 0.02	0.57 ± 0.02	
44	Hexyl tiglate	24.045	1333	0.08 ± 0.02	0.15 ± 0.01	0.77 ± 0.02	
45	Propanoic acid, 2-methyl-, butyl ester	8.682	980	0.20 ± 0.01	0.02 ± 0.01	nd	

nd—not detected; RT*—retention time, RI*—retention index (Kovat’s index); calculated retention indices are relative to n-alkanes (C10–C35).

**Table 2:** DPPH radical-scavenging activity (% inhibition) of EO vs. ascorbic acid.

Samples				Ascorbic Acid	
Concentration (mg/mL)	% Inhibition L.A.S.	% Inhibition L.A.V.	% Inhibition L.H.G.	Concentration mg/mL	% Inhibition
10	6.38 ± 0.01	8.99 ± 0.01	10.23 ± 0.01	0.006	23.81 ± 0.03
12.5	12.75 ± 0.03	13.55 ± 0.06	16.04 ± 0.06	0.008	41.73 ± 0.06
25	18.16 ± 0.13	23.01 ± 0.03	20.76 ± 0.10	0.010	55.47 ± 0.05
50	38.15 ± 0.04	37.42 ± 0.06	49.88 ± 0.06	0.014	79.16 ± 0.08
100	60.52 ± 0.07	65.78 ± 0.07	69.51 ± 0.07	0.016	91.13 ± 0.06

The values are expressed as the mean plus standard deviations of three independent determinations.

**Table 3:** Significance testing for the expression of DPPH inhibition by the ANOVA method.

ANOVA—Response					
Cases	Sum of Squares	df	Mean Square	F	*p*
EO types	374.195	2	187.097	71,126.793	<0.001
Concentration	26,210.743	4	6552.686	2.491 × 10^6^	<0.001
EO types * Concentration	281.377	8	35.172	13,371.002	<0.001
Residuals	0.118	45	0.003		

* *p* ≤ 0.05; Note. Type III sum of squares.

**Table 4:** Post hoc comparison—EO type.

			95% CI for Mean Difference					
		Mean Difference	Lower	Upper	HERSELF	t	PTukey	
L.A.S	L.A.V.	−2.557	−2.596	−2.518	0.016	−157.656	<0.001	**
	L.H.G	−6.091	−6.130	−6.052	0.016	−375.558	<0.001	**
L.A.V.	L.H.G	−3.534	−3.573	−3.495	0.016	−217.902	<0.001	**

** *p* < 0.001; Note: Value and confidence intervals are adjusted for comparing a family of 3 estimates (confidence intervals corrected using the Tukey method). Note: Results are averaged over the levels of concentration.

**Table 5:** IC_50_ values of EO samples vs. ascorbic acid.

Samples	L.A.V.	L.A.S.	L.H.G.	Ascorbic Acid
IC 50 ± SEM	4.47 ± 0.04	4.71 ± 0.04	4.10 ± 0.02	2.52 ± 0.01
R^2^	0.9015	0.9144	0.9025	0.9919
Hill Slope	13.745	13.368	15.239	17.207

Results are expressed as the mean of three determinations ± standard deviation (SD).

**Table 6:** Bacterial inhibition rates of lavender samples against ATCC strains.

L.A.V.	**mg/mL**	***S.*** ***pyogenes***	***S.*** ***aureus***	***L.*** ***monocytogenes***	***B.*** ***cereus***	***Cl.*** ***perfringens***	***P.*** ***aeruginosa***	***S. flexneri***	***E. coli***	***S*** ***typhimurium***	***H.*** ***influenzae***
	0.0075	−19.6	−22.51	−60.45	−22.28	−33.00	33.37	−6.45	−6.50	−19.37	−18.70
	0.015	−15.53	−21.86	−58.20	−20.46	−29.08	30.08	−7.80	−6.21	−17.62	−16.35
	0.03	−13.66	−18.17	−56.91	−16.04	−26.80	28.09	−9.68	−5.54	−17.57	−13.07
	0.06	−8.13	−15.59	−53.22	−18.88	−18.50	25.90	−11.24	−4.51	−11.68	−10.84
	0.125	−4.39	−13.83	−46.30	−10.76	−18.32	21.41	−13.74	−3.47	−9.03	−7.19
	0.25	−1.69	−12.06	−38.75	−4.12	−16.04	15.04	−15.71	−2.59	−3.42	−4.74
	0.5	2.83	−7.94	−28.46	5.95	−11.12	8.96	−16.55	−1.03	−1.82	−2.58
	1	14.84	2.57	−15.92	17.77	−11.06	2.59	−18.21	−0.67	9.37	−1.14
	2	23.36	5.59	−6.59	22.10	−3.61	0.50	−19.46	−0.14	21.48	−0.97
	4	32.76	17.36	0.64	31.89	4.04	−8.27	−22.16	3.25	26.85	4.60
L.A.S.	0.0075	−31.63	−14.15	−77.17	−24.10	−51.51	−4.98	−0.52	−7.61	−24.77	−24.74
	0.015	−30.50	−12.54	−69.77	−22.16	−42.27	−8.39	−0.83	−6.50	−16.11	−21.12
	0.03	−21.13	−9.97	−62.54	−21.14	−33.46	−9.86	−3.12	−5.62	−11.55	−20.77
	0.06	−14.76	−8.04	−60.77	−20.68	−24.21	−18.23	−4.99	−4.95	−10.28	−17.00
	0.125	−7.89	−6.54	−59.49	−14.56	−19.83	−19.82	−5.62	−4.29	−7.53	−14.70
	0.25	−3.03	−5.64	−44.69	−7.52	−16.48	−33.07	−6.76	−3.84	−3.97	−11.26
	0.5	−1.16	−3.86	−13.86	−3.00	−11.78	−36.35	−7.80	−3.03	−1.79	−8.74
	1	1.93	−1.88	7.88	1.16	−9.40	−40.54	−8.43	−2.29	9.24	−2.37
	2	13.81	6.80	9.00	16.40	−1.45	−45.92	−10.93	−1.33	11.83	−0.14
	4	15.56	15.27	15.27	19.25	2.42	−59.16	−12.17	0.22	22.89	3.91
L.H.G.	0.0075	−41.55	−30.70	−43.70	11.39	−35.23	40.34	7.49	−19.77	−21.98	−12.44
	0.015	−35.06	−25.31	−35.31	13.21	−26.05	37.55	6.66	−17.59	−19.15	−10.28
	0.03	−29.32	−28.36	−28.36	13.55	−20.48	33.37	4.47	−14.07	−18.20	−9.84
	0.06	−25.71	−19.32	−19.32	14.92	−17.24	32.37	3.85	−11.11	−16.97	−7.02
	0.125	−18.85	−16.24	−16.24	−16.51	−16.29	29.38	1.66	−9.37	−13.27	−5.72
	0.25	−15.74	−12.22	−11.22	−11.56	−10.48	24.80	−0.31	−7.33	−12.13	−4.69
	0.5	−12.88	−8.23	−1.23	−9.48	−8.10	21.41	−1.77	−5.62	−9.57	−2.46
	1	−8.27	−5.85	5.85	−7.18	−3.71	17.53	−3.75	−0.54	−3.39	−0.72
	2	−2.28	6.46	26.46	−3.01	5.87	2.65	−5.10	9.76	7.23	0.88
	4	6.04	15.77	31.77	6.20	17.71	8.37	−6.04	10.42	29.53	14.44

**Table 7:** LEOs (L.A.V., L.A.S., and L.H.G.) in combination with ampicillin—fractional inhibitory concentration (FIC) and FIC indices (FICI).

	MIC				MIC MIX			FIC EO			FIC Ampicillin			FICI		
*ATCC strain*	L.A.V	L.A.S	L.H.G	Amp	L.A.V	L.A.S	L.H.G	L.A.V	L.A.S	L.H.G	L.A.V	L.A.S	L.H.G	L.A.V	L.A.S	L.H.G
*S. pyogenes*	0.5	1	4	0.25	0.06	0.25	0.5	0.360	1.25	2.12	0.24	1.000	2	0.36	1.25	2.125
*S. aureus*	1	2	2	1.5	0.015	0.03	0.03	0.015	0.015	0.015	0.02	0.040	0.04	0.035	0.055	0.055
*L. monocytogenes*	4	1	1	1.5	0.06	0.125	0.125	0.015	0.125	0.125	0.04	0.083	0.083	0.06	0.21	0.21
*B. cereus*	0.5	1	4	1.75	0.03	0.125	0.015	0.060	0.125	0.004	0.017	0.071	0.009	0.08	0.2	0.01
*C. perfringens*	4	4	2	2.25	0.25	0.5	0.007	0.063	0.125	0.004	0.111	0.222	0.003	0.17	0.35	0.01
*P. aeruginosa*	2	nd *	4		0.125	0.007	0.125	0.063	0.03	0.5	nu *	nu	nu	nd	nd	nd
*S. flexneri*	nd	nd	0.125	8.25	0.007	0.015	0.125	nd	nd	1	0.01	0.020	0.01	nd	nd	1.01
*E. coli*	4	4	2	4.8	4	1	0.125	1.000	0.25	0.063	0.833	0.208	0.026	1.83	0.46	0.09
*S. typhimurium*	1	1	2	8	0.125	0.125	0.25	0.125	0.125	0.125	0.016	0.016	0.031	0.14	0.14	0.16
*H. influenzae*	4	4	1	6.5	4	0.5	0.125	1.000	0.125	0.125	0.615	0.077	0.019	1.62	0.2	0.14

* nd—not detected, i.e., the values tested had no efficacy; * nu—not usable (resistant); MIC—minimum inhibitory concentration; FIC—fractional inhibitory concentration; FICI—fractional inhibitory concentration index; FIC EO—FIC of EO = MIC of EO in combination with ampicillin/MIC of EO alone; FIC of ampicillin = MIC of ampicillin in combination with EO/MIC of ampicillin alone; FIC index = FIC of EO + FIC of ampicillin; FICI ≤ 0.5—synergistic; FICI > 0.5–1.0—additive; FICI > 1.0–4.0—no interaction; FICI > 4.0—antagonistic.

**Table 8:** Binding energies and number of bond interactions between the compounds of lavender oil and crystalised protein structures of skin bacteria (PDB: 6A9N and 8G1M).

S/No	Ligand	Binding Energy (kcal/mol)		Number of Bond Interactions	
		6A9N	8G1M	6A9N	8G1M
1	D-Limonene	−5.1	−5.5	6 alkyls	3 alkyls
2	beta-Phellandrene	−6.0	−5.6	11 alkyls	5 alkyls
3	1,8-Cineole	−6.2	−5.1	1 hydrogen	2 hydrogens, 1 alkyl
4	cis-beta-Ocimene	−5.4	−4.9	14 alkyls	7 alkyls
5	trans-beta-Ocimene	−5.5	−5.0	11 alkyls	5 alkyls
6	3-octanone	−4.9	−4.9	2 hydrogens, 4 alkyls	3 hydrogens, 2 alkyls
7	Camphor	−5.6	−5.7	1 hydrogen	1 alkyl
8	Terpinen-4-ol	−6.2	−5.4	1 hydrogen, 6 alkyls	1 C-H, 3 alkyls, 1 unfavorable Acceptor Acceptor
9	alpha-Terpineol	−6.0	−5.9	6 alkyls	3 alkyls
10	Linalyl acetate	−5.6	−4.6	2 hydrogens, 5 alkyls	2 hydrogens
11	.beta.-Myrcene	−5.1	−4.7	12 alkyls	5 alkyls
12	beta.-trans-Ocimene	−5.5	−5.0	12 alkyls	5 alkyls
13	cis-beta-Ocimene (Z)-	−5.4	−4.9	16 alkyls	6 alkyls
14	Acetic acid, hexyl ester	−5.2	−4.8	2 hydrogens, 7 alkyls	3 hydrogens, 1 C-H, 3 alkyls
15	Octen-1-ol, acetate	−5.5	−5.1	2 hydrogens, 7 alkyls	2 hydrogens, 1 C-H, 3 alkyls
16	Linalool	−4.9	−4.9	11 alkyls	2 hydrogens
17	beta.-Farnesene-	−6.2	−4.9	11 alkyls	6 alkyls
18	Caryophyllene	−6.0	−6.3	Nil	1 alkyl
19	Lavandulyl acetate	−5.4	−5.5	1 hydrogen, 1 C-H, 8 alkyls	3 hydrogens, 1 C-H, 3 alkyls
20	p-menth-1-en-8-ol	−6.0	−5.9	6 alkyls	3 alkyls
21	Nerol acetate, (Z)-	−6.3	−5.8	2 hydrogens, 6 alkyls	3 hydrogens, 1 C-H, 2 alkyls

## Data Availability

The report of the analysis performed for the samples in the paper can be found at the Interdisciplinary Research Platform (PCI) belonging to the University of Life Sciences “King Michael I of Romania”, Timisoara.
